# A Novel *Ex Vivo* Model of Aortic Valve Calcification. A Preliminary Report

**DOI:** 10.3389/fphar.2020.568764

**Published:** 2020-12-17

**Authors:** Arsenii Zabirnyk, Maria del Mar Perez, Marc Blasco, Kåre-Olav Stensløkken, Miguel D. Ferrer, Carolina Salcedo, Jarle Vaage

**Affiliations:** ^1^ Division of Physiology, Institute of Basic Medical Sciences, University of Oslo, Oslo, Norway; ^2^ Department of Research & Development, Division of Emergencies and Critical Care, Oslo University Hospital, Oslo, Norway; ^3^ Sanifit Therapeutics, Palma de Mallorca, Spain; ^4^ Institute of Clinical Medicine, University of Oslo, Oslo, Norway

**Keywords:** aortic valve, calcification, porcine, whole leaflets, *ex vivo* model, SNF472

## Abstract

**Background:** No pharmacological treatment exists to prevent or stop the calcification process of aortic valves causing aortic stenosis. The aim of this study was to develop a robust model of induced calcification in whole aortic valve leaflets which could be suitable for studies of the basic mechanisms and for testing potentially inhibitory drugs.

**Methods:** Pig hearts were obtained from a commercial abattoir. The aortic valve leaflets were dissected free and randomized between experimental groups. Whole leaflets were cultured in individual wells. Two growth media were used for cultivation: standard growth medium and an antimyofibroblastic growth medium. The latter was employed to inhibit contraction of the leaflet into a ball-like structure. Calcification was induced in the growth medium by supplementation with an osteogenic medium. Leaflets were cultivated for four weeks and medium was changed every third day. To block calcification, the inhibitor SNF472 (a formulation of the hexasodium salt of myo-inositol hexaphosphate hexasodium salt) was used at concentrations between 1 and 100 µM. After cultivation for four weeks the leaflets were snap frozen in liquid nitrogen and kept at −80 °C until blind assessment of the calcium amount in leaflets by inductively coupled plasma optical emission spectroscopy. For statistical analysis, a Kruskal–Wallis test with Dunn’s post-test was applied.

**Results:** Osteodifferentiation with calcium accumulation was in principle absent when standard medium was used. However, when the antimyofibroblastic medium was used, a strong calcium accumulation was induced (*p* = 0.006 compared to controls), and this was blocked in a dose-dependent manner by the calcification inhibitor SNF472 (*p* = 0.008), with an EC_50_ of 3.3 µM.

**Conclusion:** A model of experimentally induced calcification in cultured whole leaflets from porcine aortic valves was developed. This model can be useful for studying the basic mechanisms of valve calcification and to test pharmacological approaches to inhibit calcification.

## Introduction

Calcific aortic valve disease (CAVD) starts with fibrosis of the aortic valve leaflets and leads to calcification and aortic stenosis (AS) (Lindman et al., 2016). There is no effective pharmacological therapy for CAVD. The only treatment for AS is surgical or transcatheter aortic valve replacement. CAVD is the third leading cardiovascular disease after hypertension and ischemic heart disease and it is the most common form of valvular heart disease worldwide (Lamprea-Montealegre and Otto, 2018). The prevalence of degenerative aortic disease and CAVD increases exponentially with age (Lindman et al., 2016). In a healthy European population, 53% of people between 75 and 86 years old had signs of aortic valve calcification (Lindroos et al., 1993). Twenty-nine per cent of overall healthy persons in United States over 65 years old had aortic sclerosis and 2% had aortic stenosis (Stewart et al., 1997). In the Mediterranean area, these numbers were 73.5% and 7.4% respectively for people above 85 years (Ferreira-Gonzalez et al., 2013). The prevalence of CAVD may have a considerable increase in Europe and North America during the next 50 years due to an aging population. CAVD is also linked to the presence of other concomitant pathologies, in particular chronic kidney disease (Hensen et al., 2018) and patients treated with hemodialysis (Lin et al., 2019).

Consequently, there is an unmet need for pharmacological treatment to stop, slow, or even reverse the progression of CAVD. In order to develop such treatment, it is decisive to have good experimental models to study the cellular and molecular mechanisms of calcification as well as to test possible inhibitory agents. The most frequently used model is induced calcification in cultured aortic valve interstitial cells (VIC). These cells can be obtained from human valves and they may be crucial for understanding CAVD (Rutkovskiy et al., 2017). VIC have been extensively used to characterize aortic valve calcification including studies on inhibition of calcification (Zabirnyk et al., 2019).

Unfortunately, there is a lack of good animal models of CAVD (Sider et al., 2011; Zhang et al., 2014; Tsang et al., 2016). Although *in vitro* cell cultures are a good system to study calcification, it lacks the complexity of the valvular cell composition and extracellular matrix. Using isolated aortic valve leaflets could be a good alternative. In the model hierarchy, it brings the investigation one step up from cell cultures and into valve tissue where the interaction between cells and extracellular matrix can be additionally studied. A series of investigations have used porcine aortic valve leaflets to study the mechanical, biological or contractile properties of valve leaflet tissue (Sauren et al., 1983; Xing et al., 2004; Konduri et al., 2005; Balachandran et al., 2006; Merryman et al., 2007; Chester et al., 2008; El-Hamamsy et al., 2009; Warnock et al., 2011). Grande-Allen’s group (Swaminathan et al., 2019) used porcine valve leaflets to study how hypoxia influences extracellular matrix in the leaflets. In one study, porcine leaflets were used to investigate the effects of collagen disruption on spontaneous calcification (Rodriguez et al., 2014). Two studies induced calcification in pieces of pig leaflets. Balchandran et al. (2010) induced calcification by cyclic stretch for two weeks combined with a high concentration of osteogenic medium. Rathan et al. (2014) induced calcification in pieces of porcine aortic leaflets by adding phosphate plus inorganic pyrophosphatase. Alazarin Red was used to measure calcium in both these studies.

The aim of the present study was to develop an easy and reproducible whole leaflet model of aortic valve calcification with advanced techniques to measure the amount of calcium. Using a whole leaflet *ex vivo* instead of cell cultures will include the additional effects of the extracellular matrix. This model must be suitable for investigating the basic mechanisms of valve calcification as well as for validating inhibitory drugs as potential pharmacological therapy of calcific aortic disease.

## Materials and Equipment

### Reagents

Phosphate-buffered saline (PBS, 18,912,014, Thermo Scientific).
DMEM high glucose (41,966,052, Thermo Scientific).
Fetal bovine serum (FBS, Hyclone, SH30070.03).
Gentamicin (15,750,037, Thermo Scientific).
DMEM low glucose (31,885,023, Thermo Scientific).
Insulin (I9278, Sigma-Aldrich).
Fibroblast growth factor-2 (FGF2, SRP6159, Sigma-Aldrich).
beta-glycerophosphate (G9422, Sigma-Aldrich).
dexamethasone (D4902, Sigma-Aldrich).
ascorbic acid (A4544, Sigma-Aldrich).
SNF472 (hexasodium salt of myo-inositol hexaphosphate) (Sanifit Therapeutics, Palma de Mallorca, Spain).
1:1 nitric acid:perchloric acid mixture (Sigma-Aldrich).
Ethanol, 70% (vol/vol).

### Equipment

Scalpel
Surgical scissors
Forceps
50 ml falcon tubes.
24-well plates (Nunclon Sphera, 174,930, Thermo Scientific)
Inverted microscope
37 °C water bath
Tissue culture incubator with 37 °C and 5% CO_2_
Laminar hood
Inductively coupled plasma optical emission spectroscopy system (ICP-OES Optima 5300 DV, PerkinElmer)
Lyophilizer (VirTis Advance Freeze Dryer)

## Methods

### Whole Leaflet Cultivation

Pig hearts were obtained postmortem from a commercial abattoir, therefore no institutional ethical approval was necessary. Immediately after termination of animals, the hearts were transported on ice to the laboratory. All procedures are performed in aseptic conditions, the equipment and reagents for leaflet preparation and cultivation prior to calcium content measurement should be sterile. The culture media is prewarmed at 37 °C water bath prior use. The aortic valve leaflets were then dissected free, washed two times with PBS, randomized between experimental groups and placed in culture incubator (37 °C, 5% CO_2_) medium approximately 1 h after explantation of the heart. Whole leaflets were cultured in a separate wells in extremely low attachment cell culture 24-Well plates to avoid cell migration and leaflet integrity compromise. Two growth media were used for cultivation. Standard growth medium: DMEM high glucose, 10% FBS and 50 μg/ml gentamicin as well as an antimyofibroblastic growth medium: DMEM low glucose, 2% FBS, 50 μg/ml gentamicin, 50 ng/ml insulin, 10 ng/ml fibroblast growth factor-2. In the experimental groups where the calcification was induced the growth medium was supplemented with osteogenic medium: 10 mM beta-glycerophosphate, 0.1 µM dexamethasone and 50 µM ascorbic acid. The osteogenic media is prepared fresh prior each use. Fibroblast growth factor-2 added from frozen aliquotes to complete media immediately prior use and can not be stored at +4. Leaflets were cultivated for four weeks; medium was changed every third day.

### Experimental Groups

The leaflets were cultured for 4 weeks in either a basic growth medium or in an osteogenic medium with or without the calcification inhibitor SNF472. The following experimental series and groups were included:

Series 1 with standard growth medium
   1.1. Controls, n = 4
   1.2. With addition of osteogenic medium, n = 6
   1.3. With addition of osteogenic medium and SNF472 (30 μM, n = 6)
Series 2. Because the leaflets contracted and developed heterogenous structures with the standard growth medium, another series with antimyofibroblastic medium was added for the leaflets to keep normal configuration. This included a dose-dependent evaluation as proof of principle.
   2.1. Controls, n = 6
   2.2. With osteogenic media, n = 6
   2.3. With osteogenic medium and SNF472, 1 μM, n = 6
   2.4. With osteogenic medium and SNF472, 3 μM, n = 6
   2.5. With osteogenic medium and SNF472, 10 μM, n = 6
   2.6. With osteogenic medium and SNF472, 30 μM, n = 6
   2.7. With osteogenic medium and SNF472, 100 μM, n = 6

### Measurements of Calcium in Leaflets

After cultivation for four weeks in different growth media and supplements, the leaflets were snap frozen in liquid nitrogen and kept at −80 °C until transported on dry ice for blind assessment of the calcium amount in leaflets by inductively coupled plasma optical emission spectroscopy. Leaflets were lyophilized for 48 h and dry weight was recorded. Lyophilized tissues were digested using a 1:1 nitric acid:perchloric acid mixture in a dry bath incubator for 2–4 h at 180 °C and subsequently diluted with MilliQ™ water to a final volume of 5 ml for calcium analysis.

### Statistics

Data were analyzed by Prism 8 (Graph Pad, United States). Normality of the data was assessed using a Shapiro-Wilk test. A nonparametric one-way ANOVA (Kruskal–Wallis test) with Dunn’s post-test was applied. Graphs are shown as dot plots with median. A value of *p* < 0.05 was considered statistically significant. Half-maximal effective concentration (EC_50_) of SNF472 was calculated with a semi-logarithmic concentration response curve and a non-linear adjustment.

## Results

**FIGURE 1:**
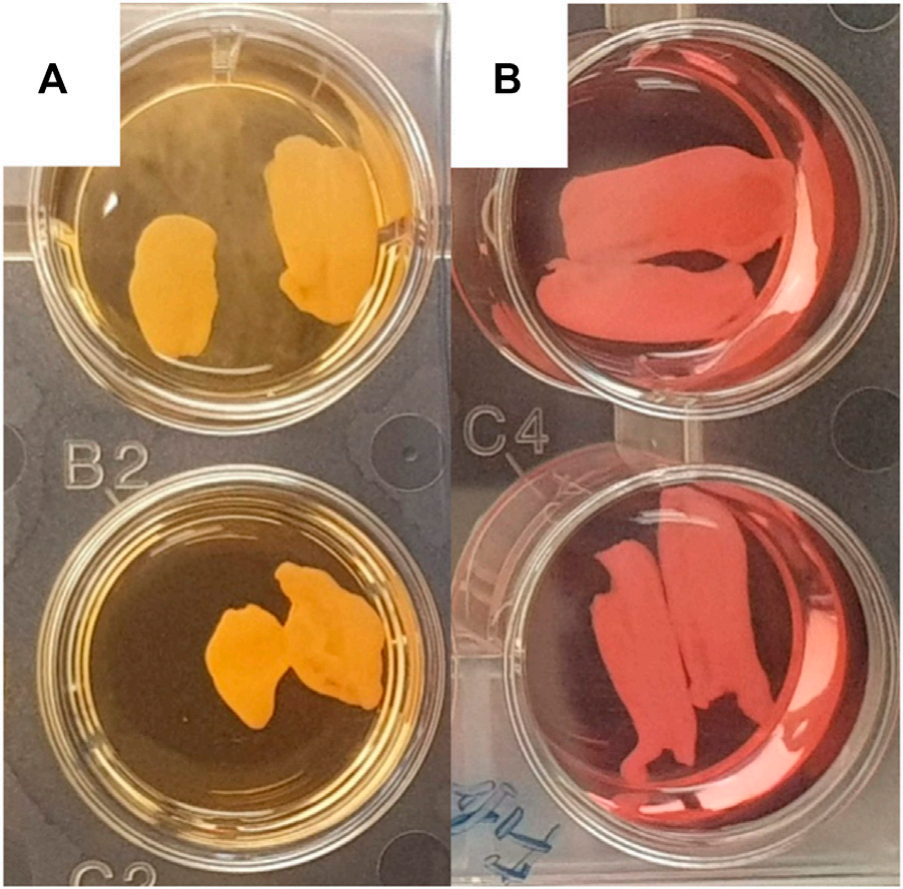
Representative pictures of wells with pig aortic valve leaflets in standard medium **(A)** and antimyofibroblastic medium **(B)**. A rounded, more ball-like appearance is apparent in leaflets in standard medium.

**FIGURE 2:**
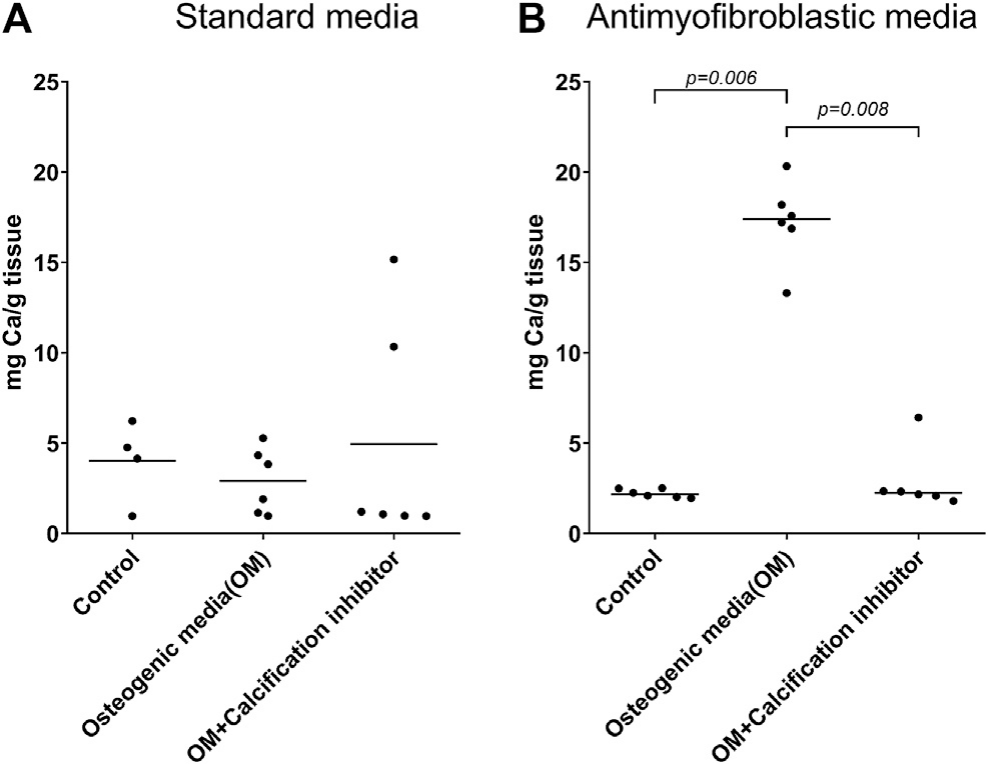
Calcium accumulation in whole pig leaflets cultivated for four weeks in standard **(A)** and antimyofibroblastic **(B)** medium under control conditions, osteogenic stimulation and osteogenic stimulation with addition of the known calcification inhibitor SNF472. The amount of calcium was measured by inductively coupled plasma optical emission spectroscopy. The data are individual values and median. Statistical analysis: nonparametric one-way ANOVA (Kruskal–Wallis test) with Dunn’s post-test.

**FIGURE 3:**
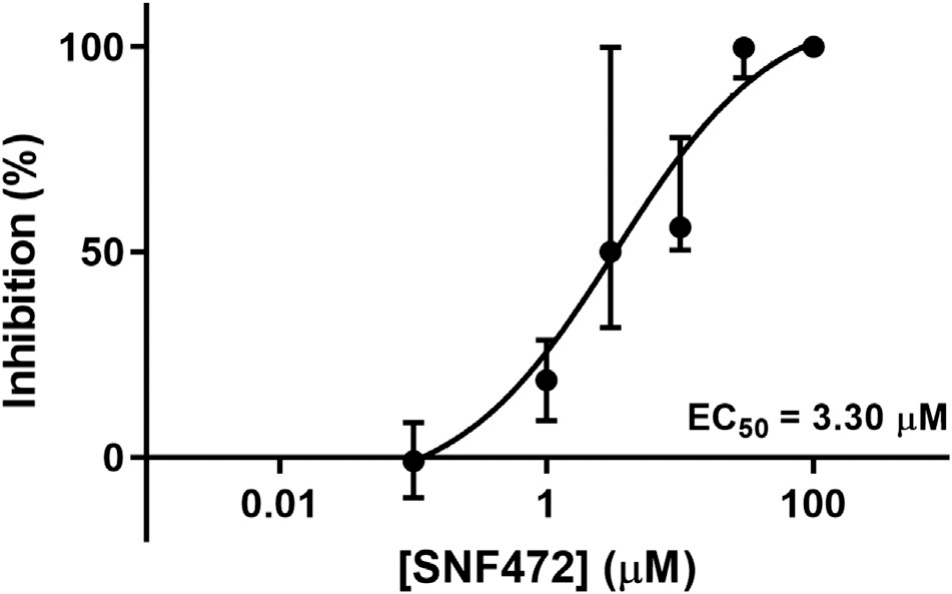
Dose-response curve of the inhibitory effects of SNF472 in whole pig leaflet calcification in antimyofibroblastic medium under osteogenic stimulation. Data is shown as median ± interquartile range.

We observed significant morphological differences between leaflets cultivated in the standard and antimyofibroblastic media: 74% of the leaflets in standard medium shrunk, become thicker than the leaflets in antimyofibroblastic medium after 2 weeks of cultivation and were more morphological diverse. They shrank to become more rounded and some developed a more ball-like shape, most probably due to myofibroblast contraction (Figure 1). A big difference occurred regarding the accumulated calcium in the cultivated leaflets: osteodifferentiation with calcium accumulation was in principle absent when standard medium was used (Figure 2A). However, when the antimyofibroblastic medium was used, a strong osteodifferentiation with considerable calcium accumulation was induced (Figure 2B). The calcium accumulation was inhibited in a dose-dependent manner and was completely blocked by 30 µM of SNF472 (Figure 2B) with an EC_50_ of 3.3 µM (Figure 3). The present results are in agreement with our previous studies on the effect of SNF472 on induced calcification in valve interstitial cell (Zabirnyk et al., 2019) as well as the finding that SNF472 significantly reduced calcification in the aortic valve in patients undergoing hemodialysis (Raggi et al., 2020). The present study did not indicate whether calcium accumulation was in the valve interstitial cells, in the extracellular matrix or both.

## Discussion

The present study showed a simple model of experimentally induced calcification in whole aortic valve leaflets *in vitro*. Furthermore, we demonstrated that such calcium accumulation could be pharmacologically inhibited in this model. Whole leaflets may be a valuable addition to the repertoire of methods to study basic mechanisms of heart valve calcification and its possible inhibition. An important observation when developing the method, was that it is necessary to use an antimyofibroblast medium to block the leaflet contraction in order to keep normal leaflet form and to obtain the calcification. This may be the reason why Balachandran et al. (2010) did not find calcification by using osteogenic medium alone. The antimyofibroblast medium is known to slow down myofibroblastic transition during cultivation of valve interstitial cells (Latif et al., 2015; Porras et al., 2017). This is especially important in the whole leaflet model preventing the leaflets from shrinking into a ball-like shape.

We have no good answer why osteodifferentiation was limited in the contracted, more ball-like leaflet in standard growth medium. Possibly, it is difficult for nutrients and the procalcification stimulus to penetrate and get access to the interior of the more spherical mass. Access to all parts of the leaflets is theoretically easier when it stays in its natural flat and thin state. It would be interesting to know why the two media had such a completely different effect on calcification. However, the main aim of the study was to create an *in vitro* model of whole porcine valve calcification, and that is achieved with the antimyofibroblast medium.

There are two other available models for inducing calcification in cultured completely aortic leaflets. Balachandran et al. (2010) cultured the cusps in a stretch bioreactor for up to two weeks in osteogenic medium supplemented with high phosphate. Mechanical stretch may partly recapitulate the clinical situation although cultured in osteogenic medium and with supplementation with phosphate. That model is more complicated requiring a bioreactor. The model by Rathan et al. (2014) have some similarities with our model. However, the calcification process induced by high phosphate went very fast (8 days). It would be interesting in the future to compare both the histopathology and the content of minerals in that model and our present model with the content in human calcified aortic valves. It might be that a slower induction of calcification may be more similar to the clinical disease regarding structure and content.

In order to induce calcification, we used osteogenic medium, with which we have extensive experience in cell cultures. This makes it possible to translate cell culture experience into the whole leaflet model. Furthermore, when studying potential inhibitors of calcification, it is important also to study if inhibitors are able to stop an ongoing calcification (Zabirnyk et al., 2019). This easier to do in a slower model.

Using a whole leaflet *ex vivo* instead of cell cultures to study the basic mechanisms of calcification itself will add information compared to standard cell cultures. The interaction between cells and the extracellular matrix is present. In the present study, we used porcine valves as material due to the simplicity of obtaining it, morphological and structural similarity to human leaflets, and sufficient amount of the tissue in each leaflet for robust analysis when comparing to leaflets from smaller animals. In general, we strongly advocate the use of human material to avoid any species differences. However, for whole leaflet studies, human material represents rather difficult logistics due to limited availability of healthy valves. For most studies on human material, material from calcified valves have been used, as they are quite easily available when explanted during surgery. However, calcified leaflets are not useful for culture of isolated leaflets to calcify; these leaflets already have a large accumulation of calcium. Nevertheless, sometimes one leaflet or parts of a leaflet of a calcified aortic valve may be without macroscopic calcification. But it is questionable if such parts or leaflets are normal or not, often they are thick and fibrotic. Normal valves are less frequently available, but may be available from donor hearts that were considered unsuitable for transplantation. Other possibilities are normal aortic valves from explanted hearts of recipients of heart transplantation. Leaflets from insufficient aortic valves may be without calcification, but such valves may be insufficient due to myomatous degeneration (Allen et al., 1985) and thus not be really “normal.” Aortic valves obtained from autopsy material as a source of cells may be possible as good cell viability has been reported up to 24 h post mortem (Gall et al., 1998). Furthermore, when using human material one should not mix genders or bicuspid and tricuspid aortic valves which have some differences in the molecular and cellular mechanisms of calcification (Aggarwal et al., 2013; Kostina et al., 2018). A disadvantage with whole leaflets from humans is that the investigator must stand ready to start the whole experiment the moment a valve is available; the leaflets cannot be frozen.

Valves from rodents like mice and rat are more different than porcine ones from human physiology and morphology (Hinton et al., 2008). Rodent valves are also smaller, more difficult to handle, and provide less material for molecular analysis. Rabbits might be an option, but it also rises a problem of the quantity of the tissue sufficient for robust analysis. We recommend that in general, porcine aortic valve from a commercial abattoir, which will be wasted anyway, is a good choice for basic studies and screening of inhibitors. It also avoids the ethical problem of obtaining human material. Human leaflets may later be used for verification.

The validity of the model is solely based on the amount of calcium accumulated in the valves. Histological characterization of the leaflets would be valuable. However, the *in vitro* leaflets would never obtain the structure of calcified human valves where the changes have developed over several years containing inflammatory cells from the blood stream and ingrowth of vasculature. To investigate the possible differences and similarities between this model and human aortic valve calcification on the molecular, cellular and histological level would be both interesting and important. However, this is beyond the scope of the present investigation. What determines the stiffness of the leaflets and causes aortic stenosis is the amount of calcification/calcium in the valve, which clinically is detected by several methods including CT scans and echocardiography. Fibrosis contributes to the stiffness of the valves, in particular in the early stages and in women (Aggarwal et al., 2013). However, it is likely that measuring calcium in the leaflets is a suitable model to test possible therapeutics for calcific aortic valve disease, although the anatomical location of calcification in the leaflet is unknown. The important matter is to have a model which works, where calcification can be induced and where potential inhibitors can be tested.

Calcification in the leaflets was strongly blocked in a dose-dependent manner by addition of the calcification inhibitor SNF472. This drug is the sodium salt of myo-inositol hexaphosphate (phytate or IP6), an endogenous inhibitor of pathological calcification (Fernandez-Palomeque et al., 2015; Ferrer et al., 2018). SNF472 acts by selective binding to the surface of hydroxyapatite crystals (Ferrer et al., 2018) and thus blocking the binding of additional calcium and phosphorus ions (Grases and Costa-Bauza, 1999; Ferrer et al., 2017) independent of the etiology of calcification. SNF472 is currently under clinical development as a treatment for calciphylaxis (Brandenburg et al., 2019) and as a treatment against the progression of cardiovascular calcification in patients receiving hemodialysis (Brandenburg et al., 2019; Raggi et al., 2020). The main potential side-effect of SNF472 is chelation of free calcium at concentrations at 100-fold its efficacy (EC50 chelation = 539 µM (Perello et al., 2020). vs EC Whole leaflets = 3.3 µM).

SNF472 has been shown to block experimentally induced calcification in cultured human valve interstitial cells with an EC_50_ of 2.02 µM (Zabirnyk et al., 2019). The present results are in line with our previous findings and the concentrations selected were based on the study of Zabirnyk et al. (2019) as well as on preliminary experiments. The concentrations are also in line with studies on inhibition of calcification in smooth muscle cells (Perello et al., 2020). The validity of the whole leaflet model to use in inhibition studies is supported by the fact that EC_50_ was quite similar to EC_50_ in cultured interstitial cells. There is no guarantee that every inhibitor of calcification may work in this model. To verify a more general action of different inhibitors is beyond the scope of the present study. The selection of SNF472 was based on the fact that the compound has alreday shown efficacy in vascular calcification in humans (Raggi et al., 2020).

To summarize, a model of culturing whole leaflets from aortic valves to induce calcification has been developed. In the model hierarchy, it is also “higher up” than cell cultures for testing potential inhibitors. This model may be useful for studying the events in extracellular matrix, cellular and molecular mechanisms of valve calcification. Its potential for investigations of potential inhibitors is evident from the dose-response curve when using SNF472.

## Data Availability Statement

The raw data supporting the conclusions of this article will be made available by the authors, without undue reservation.

## Author Contributions

AZ performed the study, developed the model, drafted part of and revised manuscript. MMP and MB performed inductively coupled plasma optic emission spectroscopy and revised manuscript. MDF and CS gave advice and revised the manuscript; KOS co-supervised and revised manuscript. JV initiated and supervised the project, drafted part of and revised the manuscript.

## Funding

AZ has been the recipient of a Scientia Fellow scholarship funded by the European Union and the Faculty of Medicine, University of Oslo, and has at present a postdoc scholarship from the Norwegian Health Association. Further funding has been received by the University of Oslo, The Norwegian Health Association, Sanifit Therapeutics and the Government of the Balearic Islands (grant number ES01/TCAI/17_2018, Conselleria d’Educació, Universitat i Recerca, with 50% charge to FEDER 2014–2020 funds). This project has been co-funded by the FEDER Program 2014-2020 of the Balearic Islands and the Government of the Balearic Islands (ES 01 / 16 2018.

## Conflict of Interest

MP, MB, and CS are employees of Sanifit Therapeutics. MP, MF, and CS are shareholders at Sanifit Therapeutics and authors of patents owned by Sanifit Therapeutics.

The remaining authors declare that the research was conducted in the absence of any commercial or financial relationships that could be construed as a potential conflict of interest.

The authors declare that this study received funding from Sanifit Therapeutics. The funder had the following involvement with the study: blindly performed calcium accumulation measurement in leaflets, did a minor revision of the manuscript.
